# Microbiota, metabolic profiles and immune biomarkers in infants receiving formula with added bovine milk fat globule membrane: a randomized, controlled trial

**DOI:** 10.3389/fnut.2024.1465174

**Published:** 2024-10-04

**Authors:** Chloe Christensen, Car Reen Kok, Cheryl L. Harris, Nancy Moore, Jennifer L. Wampler, Weihong Zhuang, Steven S. Wu, Robert Hutkins, Jacques Izard, Jennifer M. Auchtung

**Affiliations:** ^1^Department of Food Science and Technology, University of Nebraska-Lincoln, Lincoln, NE, United States; ^2^Nebraska Food for Health Center, University of Nebraska, Lincoln, NE, United States; ^3^Complex Biosystems, University of Nebraska-Lincoln, Lincoln, Nebraska, United States; ^4^Medical Sciences, Reckitt/Mead Johnson Nutrition Institute, Evansville, IN, United States; ^5^Department of Pediatrics, Indiana University School of Medicine, Indianapolis, IN, United States; ^6^Department of Internal Medicine, University of Nebraska Medical Center, Omaha, NE, United States; ^7^Frederick F. Paustian Inflammatory Bowel Disease Center, University of Nebraska Medical Center, Omaha, NE, United States; ^8^Fred and Pamela Buffett Cancer Center, University of Nebraska Medical Center, Omaha, NE, United States

**Keywords:** milk fat globule membrane, infant formula, human milk, microbiome, short chain fatty acids, clinical trial

## Abstract

**Introduction:**

Few studies have evaluated the effects of milk fat globule membrane (MFGM) on microbiota and immune markers in early infant nutrition.

**Methods:**

In this double-blind randomized study, infants (7–18 days of age) received either bovine milk-based infant formula (Control) or similar formula with an added source (5 g/L) of bovine MFGM (INV-MFGM) for 60 days. A reference group received mother’s own human milk over the same period (HM). Oral and stool samples were collected (Baseline and Day 60) to evaluate microbiota, immune markers, and metabolites.

**Results:**

At Day 60, stool bacterial diversity and richness were higher in formula groups vs HM, as were *Bifidobacterium bifidum* and *B. catenulatum* abundance. Compared to HM, stool pH was higher in Control, while acetate, propionate, isovalerate, and total short- and branched-chain fatty acids were higher in INV-MFGM. Butyrate and lactate increased for INV-MFGM from baseline to Day 60. No group differences in oral microbiota or immune markers (α- and β-defensin, calprotectin, or sIgA) were detected, although sIgA increased over time in all study groups. Added bovine MFGM in infant formula modulated stool microbiota and short- and branched-chain fatty acids compared to human milk; changes were modest relative to control formula.

**Discussion:**

Overall, distinct patterns of stool metabolites and microbiota development were observed based on early nutrition.

**Clinical trial registration:**

ClinicalTrials.gov, identifier NCT04059666.

## Introduction

The maturation of the infant gastrointestinal (GI) tract, including establishment of the GI microbiota and development of the immune system, has long-term implications on health (1). Several factors affect infant GI microbiota composition, including birth mode, environment, antibiotics, and diet (2, 3), with diet one of the most significant factors (4–6). Because of the importance of diet on infant health, the World Health Organization recommends exclusive feeding of human milk (breastfeeding) over the first six months of life to reduce risk of adverse short and long-term health outcomes for infants (7). However, a significant number of infants worldwide are fed infant formula necessitating improvements to infant formulas to more closely replicate the functions of human milk (8). Human milk components considered to have relevant functionality for incorporation and improvement of infant formula include human milk oligosaccharides, lactoferrin, and long chain poly-unsaturated fatty acids.

Evidence of the importance of milk fat globule membrane (MFGM) has increased over the past decade. MFGM is a secretory structure produced by mammary epithelial cells composed of a phospholipid trilayer that contains proteins, glycoproteins, glycolipids, and triacylglycerols (9–11). The lipid composition of human and bovine MFGM (bMFGM) is highly similar (12–15). Previous studies demonstrated MUC-1 and lactadherin, functional bMFGM components, enhance immune responses and reduce microbial adhesion within the GI tract (15, 16). In animal models, feeding formulas with added bMFGM led to significant differences in fecal microbiota (17, 18).

Addition of bMFGM could help bring the composition and functionality of infant formula closer to human milk. In infant studies, addition of bMFGM to infant formula was associated with healthy growth throughout the first year of life (19–21) and reduced susceptibility to infection (22, 23). Multiple studies demonstrated beneficial effects on neurodevelopment in infants (24–27) and young children (28). Prospective studies examining effects of bMFGM in infants showed moderate modulation of the gut microbiota (29–31) and metabolome (29–33), including increased abundance of *Bifidobacterium* species (29). bMFGM also reduced adverse health events (34) and generated serum cytokine profiles more similar to breastfed infants (35). One study found that infant formula with added bMFGM moderately affected the infant oral microbiota and reduced the presence of the common inner ear pathogen *Moraxella catarrhalis* (23). The current study was designed to investigate effects of bMFGM on development of stool and oral microbiotas and stool immune biomarkers in infants receiving a routine bovine milk-based infant formula compared to a similar formula with added bMFGM. A reference group of infants exclusively receiving mother’s-own milk was also registered.

## Materials and methods

### Study design and participants

Healthy 7- to 18-day old infants were recruited at five clinical sites in the United States (Altamonte Springs, FL; Owensboro, Ky; Birmingham, AL; Kingsport and Memphis, TN; ClinicalTrials.gov: NCT04059666)^1^ in a multicenter, double-blind, randomized, controlled, prospective trial. Participants were enrolled between March 2019 and October 2021. The study was suspended March 19, 2020 due to the COVID-19 pandemic and was re-initiated in October 2020 with an amended protocol to allow minimal in-person contact of parents and participants with the study site. The CONSORT 2010 checklist of information to include when reporting a randomized trial is included as Supplementary Figure 1. Full inclusion and exclusion criteria are described in Supplementary Table 2. COVID-19 was not assessed in participants. Parent(s) or the participant’s legal guardian(s) provided written informed consent prior to enrollment. The research protocol (protocol #3390-1) and informed consent forms observing the Declaration of Helsinki were approved by Advarra (Columbia, MD, United States; IRB #Pro00027443). Briefly, eligible infants exclusively receiving mother’s-own human milk since Day 1 of life (i.e., breastfed) with the intent to continue through the duration of the study were registered in a human milk (HM) reference group. Eligible infants whose mothers had chosen to initiate infant formula feeding and were exclusively receiving marketed infant formula for at least 24 h prior to randomization were assigned to one of two study formulas (Mead Johnson Nutrition, Evansville, IN): Control, a routine cow’s milk-based infant formula (similar to previously marketed Enfamil^®^) or investigational formula (INV-MFGM), which was similar in composition and had an added source of bMFGM (5 g/L; Lacprodan MFGM-10, Arla Foods Ingredients P/S, Denmark). Both formulas had a prebiotic blend of polydextrose and galactooligosaccharides (Table 1).

**TABLE 1:** Nutrient composition per 100 kcal (20 Calories/fl oz).

Nutrient	Study formula (target values)	
	Control	INV-MFGM
Total protein, g^a^	2.0	2.0
Total fat, g^b^	5.3	5.3
Linoleic acid, mg	780	780
α-Linolenic acid, mg	72	72
Docosahexaenoic acid, mg^b^	17	17
Arachidonic acid, mg^b^	25	25
Total carbohydrate, g^c^	11.3	11.3
Vitamin A, IU	300	300
Vitamin D, IU	60	60
Vitamin E, IU	2	2
Vitamin K, mcg	9	9
Thiamin, mcg	80	80
Riboflavin, mcg	140	140
Vitamin B6, mcg	60	60
Vitamin B12, mcg	0.3	0.3
Niacin, mcg	1000	1000
Folic Acid, mcg	16	16
Pantothenic Acid, mcg	500	500
Biotin, mcg	3	3
Vitamin C, mg	12	12
Choline, mg	24	24
Inositol, mg	24	24
Calcium, mg	78	78
Phosphorus, mg	43	43
Magnesium, mg	8	8
Iron, mg	1.2	1.2
Zinc, mg	1	1
Manganese, mcg	15	15
Copper, mcg	75	75
Iodine, mcg	15	15
Selenium, mcg	2.8	2.8
Sodium, mg	27	27
Potassium, mg	108	108
Chloride, mg	63	63

^a^Sources of protein for Control: skim milk and whey protein concentrate (WPC); and for INV-MFGM: skim milk, WPC, and whey protein-lipid concentrate (5 g/L, source of bMFGM; Lacprodan^®^ MFGM-10, Arla Foods Ingredients P/S, Denmark).

^b^Sources of fat: base blend of palm olein, soybean, coconut, and high oleic sunflower oils; fungal-derived single cell oil (source of ARA); algal-derived single cell oil (source of DHA)

^c^Prebiotic blend of polydextrose (PDX; Litesse^®^ Two Polydextrose, Danisco) and galactooligosaccharides (GOS; Vivinal^®^ GOS Galactooligosaccharide, Friesland Foods Domo). 1:1 ratio, 4g/L]) added.

The study sponsor created a computer-generated randomization schedule, provided in sealed, consecutively numbered envelopes. Each study formula was designated by its own unique code (only known by the sponsor) and assigned by opening the next sequential envelope. Formulas were provided directly to parents for the study period. Neither the product labels nor the sealed envelopes permitted unblinding by the study site. Additionally, the monitoring personnel were blinded to product identification. Blinding could only be broken in the event of a medical emergency that required knowledge of the study formula for managing the participant’s health. However, it was not necessary to break the study code prematurely in the current study. To maintain balance in enrollment of each of the study arms, it was suggested to randomize at least two infants receiving formula for each registration of an infant receiving human milk. Participants were assigned to exclusive study feeding over a 60-day period. Study visits occurred at 4 to 16 days of age (Baseline) and Day 60 (60–64 days following the start of study feeding). Researchers were blinded to the identity of the samples (identified by unique codes) until initial statistical analyses of data was completed.

### Study outcomes

The primary outcome was changes in stool microbiota. Two participant stool samples were collected by parents/caregivers at Baseline and Day 60 and returned to the study site. Samples could be obtained from more than one diaper over a 24-h period to meet the minimum amount needed for analysis: ∼0.5 g for microbial community composition (OMNIgene^®^∙GUT Collection Kit, DNA Genotek, Ottawa, Canada) and ∼5 g in a tube without stabilizer to measure pH, SCFA, and immune biomarkers. The OMNIgene^®^∙GUT Collection Kit was selected for collection of stool microbial DNA samples as previous studies had demonstrated sample stability at room temperature (36). After collection, samples were stored at 4°C until transported the study site, at which point they were stored at −20°C. Samples were shipped on dry ice to the University of Nebraska-Lincoln and subsequently stored at −80°C until further processed.

Secondary outcomes included oral microbiota; stool color, consistency, pH and SCFA; 24-h formula intake recall; and medically-confirmed adverse events. Buccal swabs were collected at Baseline and Day 60 by study site personnel using a specialized swab and tube (OMNIgene∙ORAL Collection Kit, DNA Genotek). Participants had not eaten within 30 min prior to collection. All samples were stored at a minimum of −20°C at the study site, shipped on dry ice to the University of Nebraska-Lincoln, and subsequently stored at −80°C until further processed.

Body weight was recorded at Baseline. Parents completed a 24-h recall of study formula intake (fluid oz/day) by phone at Day 30 (± 5 days) and at the Day 60 study visit. A 48-h recall of stool consistency (responses scaled from 1 to 5: hard, formed, mushy, unformed or seedy, watery) was collected at Baseline, Day 30, and Day 60. Adverse events (categorized as: Body as a Whole; Eyes, Ears, Nose, and Throat; Gastrointestinal; Metabolic and Nutrition; Musculoskeletal; Respiratory; and Skin) were recorded throughout the study.

### Community sequencing and analysis

DNA from stool and oral samples was extracted using a previously-described bead-beating phenol-chloroform method (37). 16S rRNA gene sequencing of the V4 variable region was performed as described previously (38). Initial sequence analysis was conducted with DADA2 (39) in R (ver. 4.2.1). Forward and reverse reads were truncated to 240 and 210 bp, respectively. Sequences were de-replicated into unique amplicon sequence variants (ASV) and a list of representative sequences with 1,484 features was generated. Taxonomy was successfully assigned to 1,359 features using the SILVA database (40) (release 1.38.1) with a classifier based on 99% sequence identity, filtering out Archaeal, Chloroplast, or Mitochondrial sequences. The raw 16S rRNA sequences were deposited in the NCBI database under BioProject ID PRJNA1005334.^2^

### pH and S/BCFA

Fecal samples were diluted (1:10, deionized water) and homogenized prior to pH measurement as described (37). Short chain fatty acids (SCFA; acetate, butyrate, propionate) and branched-chain fatty acids (BCFA; isovalerate and isobutyrate) were measured using gas chromatography as described and are reported per wet weight of stool (37). To measure lactic acid, stool samples were homogenized, diluted 1:10 in 10 mM sulfuric acid and filtered through 0.45 μm filters. Samples (10 μL) were injected into a HPLC (Aligent 1260 Infinity, Waldbronn, Germany) containing Aminex HPX-87H column (300 x 7.8 mm, Bio-Rad) equipped with a diode array detector. The column was held at 50°C with a 0.6 mL/min flow rate; 10 mM sulfuric acid was the mobile phase. Lactic acid concentrations were calculated relative to a standard curve.

### Immune biomarkers

Enzyme-linked immunosorbent assay (ELISA) kits were used to determine stool secretory immunoglobulin A (sIgA), β-defensin, and calprotectin (Immunodiagnostik AG, Bensheim, Germany) and α-defensin (Hycult Biotech, Wayne, PA USA). Assays were performed according to manufacturers’ protocols on two biological replicates in technical duplicate.

### Statistical analyses

The sample size was chosen to investigate stool microbiota composition as the primary variable in infants receiving one of two study formulas or mother’s-own breast milk for a 60-day feeding period. Specifically, group differences in infant stool microbiota between human milk and formula fed infants had been previously demonstrated in studies with three groups of 20 participants (37). Group differences in children’s stool microbiota were also observed between participants that differed in the presence or absence of diabetes-associated autoantibodies with two groups of 18 participants (41). To have a total of 20 infants per group with protocol-compliant samples at Baseline and Day 60, the planned sample size for the study was originally 111 participants (37 per group; allowing for a potential 45% drop-out rate). However, enrollment was interrupted and subsequently complicated by the COVID-19 pandemic. As a result, the study concluded prematurely and the final sample size was reduced. Nevertheless, sample sizes were sufficiently powered to observe differences in stool microbiota at Day 60 between HM and formula feeding groups. Differences in subject age at baseline was analyzed by Kruskal-Wallis test with Dunn’s correction for multiple comparisons. Formula intake was analyzed by ANOVA. Stool consistency was analyzed using the Cochran-Mantel-Haenszel row means score test. Incidence of adverse events was analyzed using Fisher’s exact test. Unadjusted pairwise comparisons were performed if the overall test was statistically significant. These tests were conducted at α = 0.05 using SAS version 9.4 (Cary, NC), except for differences in subject age at baseline, which was performed with Graphpad Prism 10.2 (Boston, MA).

Statistical analysis of 16S rRNA gene sequencing was performed in R (ver. 4.1.0 and 4.3.1). Shannon and Observed richness indices were computed at the ASV level. Pairwise comparisons of Baseline and Day 60 variation in each group, and between group variation at Day 60 were performed through Kruskal-Wallis testing. Holm FDR correction was integrated, and significance was determined at α = 0.05. No significant differences in Observed ASV richness were observed between study groups at Baseline. Data was plotted with GraphPad Prism (ver 9.5). Bray-Curtis, Binary Jaccard, and Unweighted UniFrac dissimilarities were calculated on relative abundance data and visualized with Principal Coordinates Analysis plots (PCoA), with significance of differences determined by pairwise permutational multivariate analysis of variance (PERMANOVA) with Bonferroni FDR correction.

Prior to assessing taxonomic differences, features were filtered to remove those detected in a single sample or in the lowest 10% of sequences, for a total of 270 features. Taxa abundance was compared by Wilcoxon sign rank testing with Benjamini-Hochberg FDR correction on log_2_-transformed data; heat maps were generated using Metacoder (42) for genera with > 10 reads and significant differences detected. Significantly different ASVs were also identified by comparing Baseline to Day 60 and between groups at Day 60 using DESeq2 (43). To account for high positive fold-changes present in low abundance features, ASVs with significant differences but a mean abundance < 100 reads ( < 0.25% relative abundance) were removed. Significant differences in *Bifidobacterium* species abundance were determined by ANOVA with Brown and Forsythe correction for unequal variance on log_2_-transformed data floored at minimum relative non-zero abundance using GraphPad Prism (ver 9.5).

Changes in pH, S/BCFA, and immune biomarkers were analyzed using SAS ver. 3.8; all tests were conducted with α = 0.05. Least square means (LSM) of fixed effects (pH, S/BCFA, and immune biomarkers) for each group between Baseline and Day 60 with a repeated measure ANOVA were compared using a Toeplitz covariance structure. Bonferroni adjustment was used for multiple-comparison correction. For this model, ‘groups’ and ‘visits’ were the fixed effects while ‘visit’ was the repeated measure, and subject within group was the assigned random error. To assess group differences, analysis of covariance (ANCOVA) was performed, with baseline measurements assigned as the covariate. Group differences between the LSM of Day 60 measurements were compared. Double square root transformations were applied to all pH, S/BCFA, and immune biomarkers to adjust for normality. An analysis of probability of a zero observation was performed on measurements of isobutyrate and isovalerate, resulting in a modified log transformation used to adjust for normality after the removal of zeros from the dataset. Data was plotted with GraphPad Prism (ver 9.5).

## Results

### Participant characteristics

**FIGURE 1:**
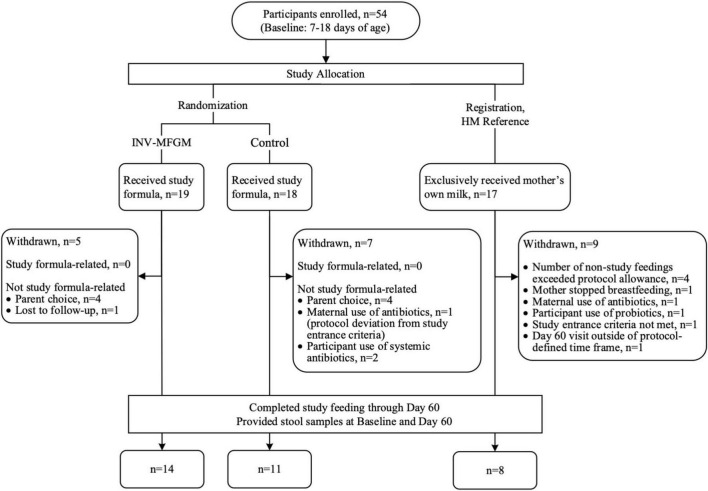
Study allocation.

A total of 54 participants were enrolled in the study (Figure 1); 33 participants completed the study (Control: n = 11; INV-MFGM: n = 14; HM: n = 8). Infants were 37 to 42 weeks gestational age at birth and all were delivered vaginally. Mothers of eligible infants had chosen to initiate formula feeding prior to study enrollment, and infants in formula feeding groups had exclusively received infant formula for at least 24 h prior to randomization. Supplementary Table 2 lists full inclusion and exclusion criteria. No differences in weight (mean ± SE) at birth or study entry, sex, race, or ethnicity were detected (Table 2). No significant differences between study groups were observed in days of age when baseline samples were collected (Supplementary Table 3). No group differences were detected in parent-reported study formula intake (mean fluid oz/day) at Days 30 and 60. At Day 30, control and INV-MFGM had an intake of 27.6 ± 1.5 oz and 26.3 ± 1.3 oz, respectively (p = 0.544). Similarly, at Day 60, control and INV-MFGM had an intake of 29.8 ± 1.7 oz and 29.2 ± 1.5 oz, respectively (p = 0.792). For stool consistency, no group differences were detected at Baseline (Supplementary Table 4). At Day 30 significant differences between Control and HM groups were detected; primary differences were more infants with stool categorized as “mushy” in the Control and stool categorized as “unformed or seedy” in the HM group. At Day 60, significant differences in distribution included more infants in Control and INV-MFGM groups with “mushy” stool vs more infants in the HM group with “unformed or seedy” or “watery” stool. No group differences were detected in the number of participants with at least one adverse event reported (Control: n = 8, 44%; INV-MFGM: n = 13, 68%; HM: n = 6, 35%; p = 0.131). Incidences of adverse events (categorized as: Body as a Whole; Eyes, Ears, Nose, and Throat; Gastrointestinal; Metabolic and Nutrition; Musculoskeletal; Respiratory; and Skin) were low with no significant differences by group. No serious adverse events were reported. No significant differences in study discontinuation were detected and no study discontinuations were related to study formula.

**TABLE 2:** Infant characteristics at birth and study entry.

	Study group			*P*
Characteristic	Control (*n* = 18)	INV-MFGM (*n* = 19)	HM (*n* = 17)	
**At Birth**				
Weight^a^ (g)	3442.0 ± 106.3	3385.5 ± 103.3	3547.4 ± 109.7	0.558
Sex (number of females/males)	8/10	10/9	10/7	0.700
**Race^b^, *n* (%)**				
White	14 (78)	17 (89)	16 (94)	0.645
Black	1 (6)	1 (5)	0 (0)	
Other	3 (17)	1 (5)	1 (6)	
**Ethnicity**				
Hispanic	0 (0)	1 (5)	1 (6)	0.761
Not hispanic	18 (100)	18 (95)	16 (94)	
Weight at study entry (g)^a^	3535.3 ± 109.1	3550.4 ± 106.1	3791.5 ± 112.6	0.198

^a^Mean ± standard error (SE)

^b^The case report form provided 7 categories in which to record an infant’s race: White, Black or African American, Asian, Native American/Alaskan Native, Native Hawaiian or Pacific Islander, More than one race, and Unknown, or not reported. Because of the small number of participants in the Native Hawaiian/Pacific Islander and More than one race categories, these categories were combined to form an Other category for analysis. The analysis did not include the category Unknown or not reported.

### Effects of infant feeding duration and type on microbial community structure

**FIGURE 2:**
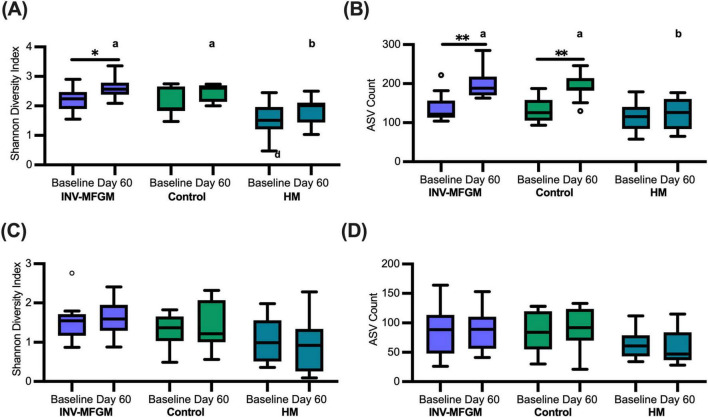
Diversity and richness of microbiotas. For stool **(A,B)** and oral microbiotas **(C,D)**, pairwise comparisons of **(A,C)** Shannon diversity and **(B,D)** ASV richness were performed by study time point (Baseline and Day 60) and feeding group. Boxplots show the median, first and third quartiles with the whiskers extending to 1.5 x IQR (Interquartile Range). ° Used to indicate points outside of whiskers. Unique letters above Day 60 boxplots indicate significant difference between study groups. *p < 0.05; **p < 0.01.

Overall changes in stool and oral microbiota within and between study groups were assessed (Figure 2). No differences in stool richness (Observed amplicon sequence variants (ASVs) or diversity (Shannon Diversity) were detected at Baseline. Richness and diversity for the HM group remained stable across the feeding period, whereas richness increased significantly from Baseline to Day 60 in both formula groups and Shannon diversity increased significantly in the INV-MFGM group. Diversity and Richness were significantly higher for both INV-MFGM and Control compared to the HM group at Day 60. For oral microbiota, no group differences in diversity and richness were detected at Baseline or Day 60 or from Baseline to Day 60.

To assess overall differences in community composition, Bray-Curtis, Jaccard, and UniFrac dissimilarities were calculated and visualized using PCoA (Supplementary Figure 1). For stool, there were no significant differences between study groups based on phylogenetic diversity (UniFrac). For comparisons based upon ASV composition (Jaccard), both formula groups were significantly different from the HM reference group, although the magnitude of differences was low (INV-MFGM: R^2^ = 0.089, p = 0.003; Control: R^2^ = 0.120, p = 0.003) and there were no significant differences between formula groups. A small amount of variation was observed between INV-MFGM and HM reference groups (R^2^ = 0.083, p = 0.030) based upon ASV abundance (Bray-Curtis), but no other differences between study groups were significant. For oral microbiota, there were no significant differences in phylogenetic diversity or composition between study groups; small differences in overall ASV abundance were observed between INV-MFGM and HM groups (R^2^ = 0.134, p = 0.039). Spearman correlation performed on amplicon sequence variants (ASVs, equivalent to unique species or strains) with > 0.25% abundance identified 22 ASVs conserved across oral and stool (Supplementary Figure 2).

### Composition of infant stool and oral microbiotas

Relative abundance of stool (Supplementary Figure 3) and oral (Supplementary Figure 4) genera at Baseline and Day 60 varied across individuals. Abundance of *Bifidobacterium* in stool microbiota varied widely by individual and study time point. Oral microbiotas were dominated by two main genera, *Streptococcus*, present in all samples at Baseline and Day 60, and *Veillonella*, which was more common at Day 60.

**FIGURE 3:**
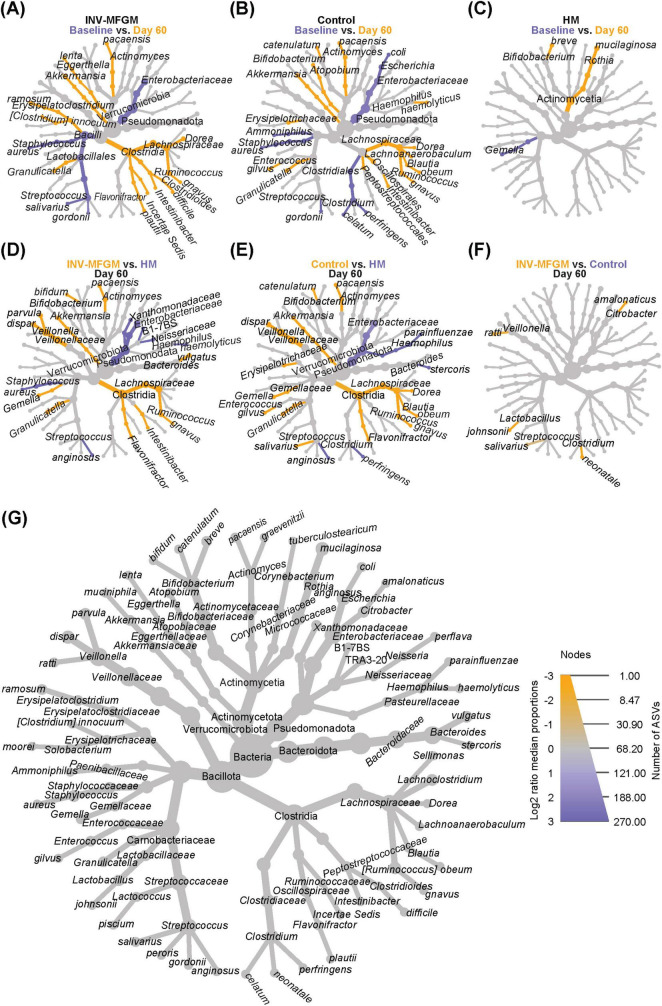
Differential heat trees comparing infant stool taxa. Difference in relative abundance by study time point (Baseline vs. Day 60) shown by feeding group: **(A)** INV-MFGM, **(B)** Control, and **(C)** HM. Differences in relative abundance between study groups at Day 60 shown by comparisons of: (**D**) INV-MFGM vs HM, **(E)** Control vs HM, and **(F)** INV-MFGM vs Control. **(G)** A reference taxonomic tree representing all taxa with differential abundance and key indicating the correlations between node size and total ASVs and color intensity with relative abundance.

Heat trees were used to visualize differences in stool microbiota taxa for each study group between Baseline and Day 60 (Figures 3A–C) and between study groups at Day 60 (Figures 3D–F). In both formula groups, *Enterobacteriaceae* and *Streptococcus* spp. decreased from Baseline to Day 60, while *Dorea, Ruminococcus gnavus, Intestinabcter, Granulicatella, Akkermansia* and *Actinomyces paecansis* increased. In the HM group, *Rothia mucilaginosa* and *B. breve* increased from Baseline to Day 60, whereas *Gemella* decreased. At Day 60, formula groups had higher *Ruminococcus, Flavonifractor, Granulicatella, Gemella, Veillonella, Bifidobacterium*, *Akkermansia*, and *Actinomyces* and lower *Enterobacteriaceae, Haemophilus*, and *Streptococcus anginosus* compared to HM. Higher *Intestinibacter* and *Bacteroides* and lower *Staphylococcus, Xanthomonadaceae* and *Burkholderiales* were detected for the INV-MFGM vs HM group, whereas higher *Erysipelotrichaceae, Enterococcus, Streptococcus salivarus, Blautia*, and *Dorea* and lower *Bacteroides stercoris* were detected for the Control vs HM group. Few differences were detected between formula study groups. Compared to the HM group, different ASVs that classify as *Bifidobacterium* at the species-level were enriched in each study formula group: *B. bifidum* in the INV-MFGM group and *B. catenulatum* in the Control group. Few group differences for oral communities at Day 60 were detected (Supplementary Figure 5).

**FIGURE 4:**
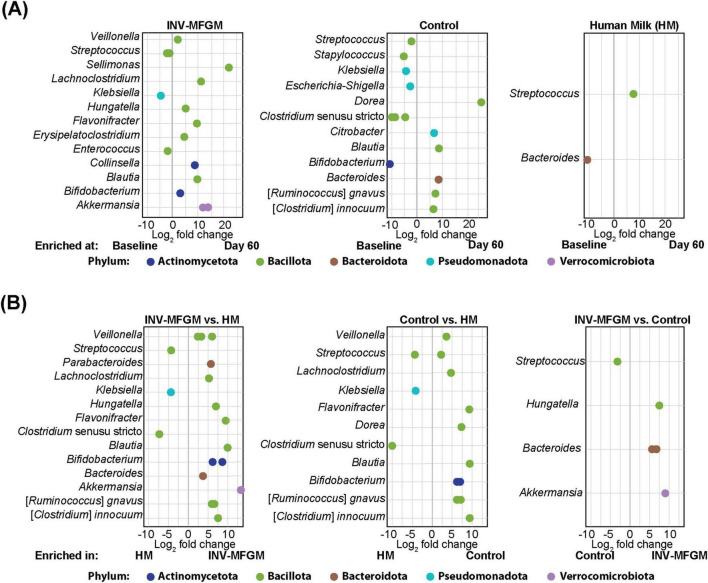
Analysis of differentially abundant infant stool ASVs. ASVs with differential abundance between **(A)** Baseline and Day 60 and **(B)** study groups at Day 60.

**FIGURE 5:**
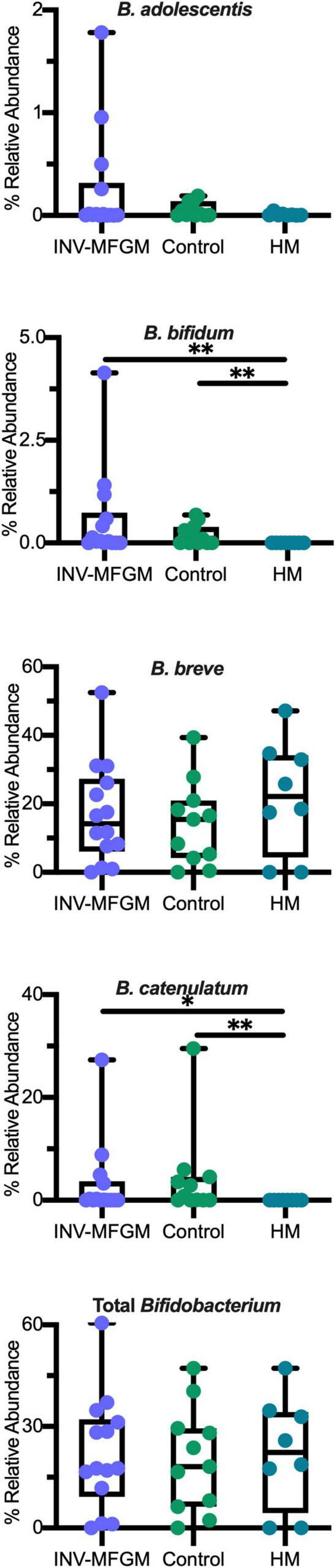
Differences in relative abundance of individual *Bifidobacterium* species and total *Bifidobacterium* between study groups at Day 60. Boxplots show the median, first and third quartiles with the whiskers extending through minimum and maximum. *p < 0.05; **p < 0.01.

DESeq2 analysis was used to compare differences in ASV abundance in stool between Baseline and Day 60 for each study group (Figure 4A) and between study groups at Day 60 (Figure 4B). At Day 60, higher *Veillonella, Lachnoclostridium, Flavonifractor, Blautia, Bifidobacterium, Ruminococcus*, and *Clostridium innocuum* and lower *Streptococcus, Klebsiella*, and *Clostridium* sensu stricto were detected in formula groups vs HM. Compared to the Control, the INV-MFGM group had higher *Hungatella, Bacteroides*, and *Akkermansia* ASVs, and lower *Streptococcus*. *B. catenulatum* and *B. bifidum* were higher in both study formula groups vs HM. Wilcoxon rank sum tests were performed to investigate differences in abundance of the *Bifidobacterium* genus and species between study groups at Day 60 (Figure 5). No differences in abundance of the *Bidobacterium* genus and the most abundant *Bifidobacterium* species, *B. breve*, were detected between study groups. Significantly higher *B. catenulatum* and *B. bifidum* were detected in formula groups compared to HM.

For oral microbiotas, *Staphylococcus* decreased across study groups from Baseline to Day 60 (Supplementary Figure 6A). Study formula groups also demonstrated increased *Granulicatella* and *Alloprevotella* levels and decreased *Streptococcus* from Baseline to Day 60. At Day 60 (Supplementary Figure 6B) both formula groups had increased *Prevotella*, *Porphyromonas*, *Granulicatella* and *Lachnoanaerobaculum* compared to HM; *Alloprevotella* was higher for INV-MFGM compared to HM; and *Rothia* and *Veillonella* were higher for the Control group compared to HM. There were no significant differences between formula groups at Day 60.

### Stool pH and S/BCFA

**FIGURE 6:**
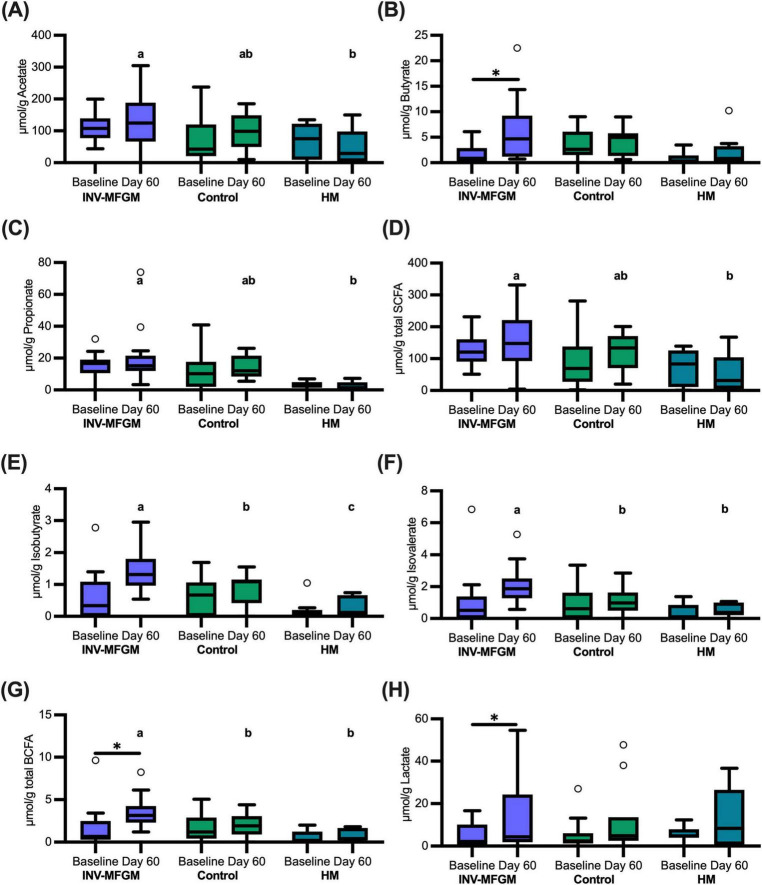
Stool S/BCFA and lactate concentrations. **(A)** Acetate, **(B)** butyrate, **(C)** propionate, **(D)** total SCFA, **(E)** isobutyrate, **(F)** isovalerate, **(G)** total BCFA, and **(H)** lactate were measured at Baseline and Day 60 and were reported as μmol/g of wet weight stool. Boxplots show the median, first and third quartiles with the whiskers extending to 1.5 x IQR (Interquartile Range). ° Used to indicate points outside of whiskers. Unique letters above Day 60 boxplots indicate study groups with significantly different values. *p < 0.05.

**FIGURE 7:**
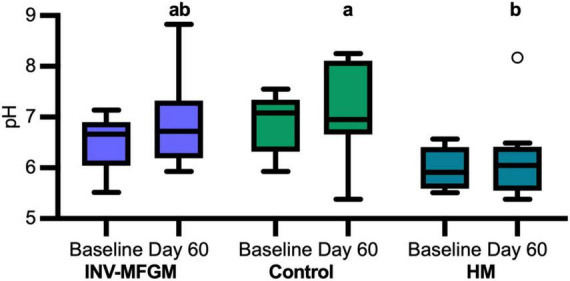
pH of infant stool. pH was measured at Baseline and Day 60. Boxplots show the median, first and third quartiles with the whiskers extending to 1.5 x IQR (Interquartile Range). ° Used to indicate points outside of whiskers. Unique letters above Day 60 boxplots indicate study groups with significantly different values.

Stool S/BCFA are shown in Figure 6. No significant group differences in S/BCFA were detected at Baseline, with the exception of significantly lower propionate in the HM vs INV-MFGM group. Butyrate, total BCFA, and lactate significantly increased in the INV-MFGM group from Baseline to Day 60. Acetate, propionate and total SCFA were significantly higher in the INV-MFGM vs HM group at Day 60. Total BCFA and isovalerate were significantly higher for INV-MFGM vs Control and HM groups at Day 60 and isobutyrate was significantly different among groups (INV-MFGM > control > HM). No correlations between stool genera and metabolites were detected. No group differences in stool pH were detected at Baseline or from Baseline to Day 60 (Figure 7). However, stool pH was significantly higher for the control vs HM group at Day 60.

### Immune biomarkers

**FIGURE 8:**
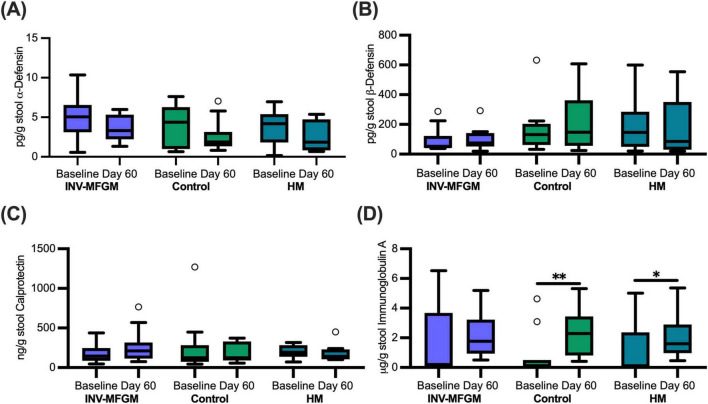
Infant stool immune biomarkers. Concentrations of **(A)** α-defensin, **(B)** β-defensin, **(C)** calprotectin, and **(D)** secretory immunoglobulin A measured at Baseline and Day 60. Boxplots show the median, first and third quartiles with the whiskers extending to 1.5 x IQR (Interquartile Range). ° Used to indicate points outside of whiskers. No significant differences were observed between groups at Day 60. *p = 0.03; **p = 0.01.

Immune biomarkers are shown in Figure 8. No group differences were detected for α-defensin, β-defensin, and calprotectin at Baseline or Day 60 or between Baseline and Day 60. No significant group differences were detected at Baseline or Day 60 for sIgA; however, sIgA concentrations increased from Baseline to Day 60 for Control and HM.

## Discussion

The effects of MFGM on stool and oral microbiota and immune markers in early infant nutrition were investigated in healthy infants receiving routine cow’s milk-based infant formulas with or without added bovine MFGM. This study complements the growing foundation of clinical data supporting the safety, tolerability, and potential functional benefit of adding bMFGM to infant formula (19–23). Multiple studies have also demonstrated beneficial effects on behavior and neurodevelopment in infants (24–27) and in young children through 5.5 years of age (28). The current study extends the body of clinical data supporting MFGM to the earliest period of nutrition in infancy. *B. bifidum* and *B. catenulatum* species were higher in infants receiving study formulas compared to a reference group receiving mother’s-own milk. In addition, distinct patterns of stool S/BCFA were observed based on study feeding.

These results suggest one or more components of bMFGM could be metabolized by the digestive tract microbiota, leading to modulation of S/BCFA. An earlier study that examined infant gut maturation reported higher stool isobutyrate, isovalerate, and propionate in infants receiving bMFGM in formula compared to breastfed infants (30). In the current study, similar results were observed, as well as higher acetate and total S/BCFA in the INV-MFGM group compared to HM. Differences in isobutyrate were also detected in study formula groups and higher isovalerate and total BCFA were observed in the INV-MFGM vs Control and HM groups. While variations in stool consistency between HM and formula-fed groups may contribute to differences in total branched and short chain fatty acids measured in stool normalized to wet weights, the effect of stool consistency is likely to be modest based on previous correlations between stool consistency and water content (44). We previously reported feeding extensively hydrolyzed protein formula led to higher stool BCFA in infants as well as higher stool pH (37). In this study, stool pH values in infants receiving Control formula were 0.9 pH units higher compared to infants receiving mother’s own milk, while pH was not significantly different in infants fed formula with added bMFGM compared to infants fed mother’s own milk. In addition to production of BCFA, protein fermentation can lead to increased production of amines which can increase pH (45). Alternatively, differences in stool pH between Control and HM groups could be due to HMO fermentation in the HM group which lowers pH (46). Further studies would be needed to determine whether these differences in stool pH are significant and what mechanism(s) potentially contribute to these differences.

Richness and diversity in stool was significantly higher in infants receiving either study formula compared to HM at Day 60, similar to previous reports (37, 47, 48). High diversity in the GI microbiota across the majority of the lifespan is generally considered to reflect a healthier state (49). However, low diversity is considered to reflect a healthier state in infants, as low diversity is often due to enrichment of HMO-consuming *Bifidobacterium* taxa (50). As no differences in overall *Bifidobacterium* levels between groups were detected in our study, it is unclear how these differences in microbiota richness and diversity would affect function.

The primary stool taxa detected across all groups and timepoints belonged to phyla typically associated with the infant gut microbiota (51, 52). Consistent with previous studies (51, 53, 54), *Bifidobacterium* was abundant for many participants. However, *Bifidobacterium* appeared to be absent or below detection for several participants at Baseline and Day 60 in all study groups. The absence of *Bifidobacterium* in infants has been observed previously (55, 56) and may be due to several factors, including mode of delivery (57, 58), normal daily fluctuation of *Bifidobacteria* in stool, founder effects, and displacement by other taxa (59).

Infant-associated *Bifidobacterium* usually include *B. breve, B. bifidum* and *B. longum* (60, 61). While we observed *B. breve* and *B. bifidum*, we also observed *B. adolescentis, B. catenulatum*, and *B. dentium*. *B. breve* was the most abundant species detected in all groups. *B. catenulatum* and *B. bifidum* were higher in both formula groups compared to the HM group. Although *B. catenulatum* has been associated with adult microbiotas (62), some strains also appear in the gastrointestinal tract of infants (63), especially post-weaning (64). A subspecies of *B. catenulatum, B. catenulatum* subsp. *kashiwanohense*, is found to primarily colonize infants and encodes genes for metabolism of human milk oligosaccharides (HMO) (63). *B. bifidum* has been reported to colonize infants via maternal transfer and to have co-evolved with the human host (65). The ability of *B. bifidum* to efficiently degrade complex glycans, such as those found in human milk oligosaccharides (65), and as plant-associated carbohydrates (66), allow *B. bifidum* to enhance the growth of other *Bifidobacterium* species through cross-feeding. The absence of *B. longum* (particularly *B. longum* subsp. *infantis*) in the HM group is consistent with previous observations that this subspecies with unique abilities to consume HMOs is often absent in infants receiving human milk in resource-rich countries (55). However, accurate species-level identification using the V4 region is challenging which could have resulted in unclassified *Bifidobacterium* at the species level (67).

The inclusion of a prebiotic blend of polydextrose and galactooligosaccharides likely contributed to the enrichment of *Bifidobacterium* taxa in formula-fed infants as has been previously reported (68). A previous study that observed enrichment of *Bifidboacterium* taxa in response to supplementation of formula with bovine MFGM compared to control formula did not appear to contain prebiotics (29). In contrast, a second study where formula composition was more similar to that described here (supplemented with galactooligosacchardes at 2.3 g/100g) did not observe significant differences in *Bifidobacterium* between study groups fed formula with or without bovine MFGM (30). Neither study reported change in *Bifidobacterium* species; rather differences were reported for the *Bifidobacterium* genus.

Previous reports suggest lower stool α- and β- defensin concentrations in infants receiving human milk vs standard formula (69). In contrast, higher stool calprotectin and sIgA were reported in infants receiving HM compared to exclusive formula or mixed feeding (69, 70). Immune biomarkers concentrations in this current study were within the ranges reported in previous studies, but no group differences were detected at Baseline or Day 60. While increased sIgA concentrations from Baseline to Day 60 for Control and HM groups were detected, there were no group differences detected either at Baseline or Day 60. Lack of differences between study groups at Day 60 is consistent with a previous study assessing effects of consumption of MFGM-supplemented formulas which found no significant differences in fecal sIgA levels in infants that were consuming formula with and without MFGM supplementation at 4 months of age (19).

The few studies to consider effects of diet on the infant oral microbiota have suggested breastfeeding reduces bacterial diversity (71–73). However, no differences in diversity from Baseline to Day 60 or between study feeding groups at Day 60 were detected in the current study. Inability to detect differences in oral microbiota diversity between study feeding groups may be due to small sample sizes. Previous studies that demonstrated significant differences between breastfed and formula fed infants or between infants consuming standard formula and formula supplemented with MFGM have had much larger numbers of participants, although one study observed significant differences between breastfed and formula fed infants in a similarly small study. Consistent with this hypothesis, statistical significance of differences in Day 60 ASV were detected between HM and INV-MFGM (p = 0.138) and HM and Control p = 0.137). The primary bacterial taxa detected across all study time points and feeding groups belong to phyla typically associated with the infant oral microbiota (74). Consistent with previous studies (74–76), *Streptococcaceae* were dominant members of the oral microbiota. No species were identified as cariogenic *Streptococcus mutans*, which has been associated with negative impacts on oral and overall health (77). Although a total of 22 ASVs correlated between oral and fecal microbiotas, the oral-gut microbial axis remains an area of interest not extensively studied in infants (76).

Results from this study suggest that formulas with different lipid ingredients affect the microbiota differently. Compared to previous studies, this study limited the impact of covariables such as use of probiotics, prebiotics, or other immune-modulating ingredients in addition to bMFGM. Other key strengths of this study include enrollment of infants at a very early age, inclusion of a human milk reference, and strict entry criteria which excluded Caesarian section born infants or those who received antibiotics or probiotics peri-partum. These criteria reduced potential variability that could be introduced by these environmental factors. Limitations of the study included small sample size due to difficulty in recruitment following the outbreak of the worldwide COVID pandemic. While having the planned sample size would have been ideal, the data reported here is valuable in demonstrating the development of oral and stool microbiota populations in infants. Data on infant feeding history (such as breastfeeding or use of infant formula prior to randomization), materinal pre-gestational health, and perinatal antibiotic use may also have provided further insights into variation between study groups at baseline. Further studies with a larger sample size, that include both Caesarian-section and vaginal births to evaluate effects of real-world practices, and longitudinal studies that examine both microbiota and physiological outcomes may be needed to fully understand the potential systemic benefits that result from the incorporation of functional components in infant formulas.

## Conclusion

Infants exclusively receiving a routine cow’s milk-based infant formula with or without added bMFGM or mother’s own milk from early infancy (7–18 days of age) had distinct patterns of gut and oral microbiota development over a 60-day feeding period. *Bifidobacterium* abundance was generally similar across study feeding groups, with *B. breve* the dominant species. *B. bifidum* and *B. catenulatum* were enriched in formula-fed groups. Additionally, *Akkermansia* species were higher in infants receiving bMFGM in formula compared to other groups consistent with a previous report on bMFGM supplementation (30). Although some health-associated taxa were identified in infants receiving bMFGM in formula, microbiota composition was generally most similar between formula groups compared to infants receiving mother’s own human milk. In addition, infants in the HM group had consistently lower stool pH and microbial metabolite concentrations. Infants receiving added bMFGM in formula had significantly higher butyrate and lactate at Day 60 compared to baseline and significantly higher BCFA compared to control formula at Day 60. Microbial metabolite profiles were also differentiated by study feeding group, however, further studies are needed to determine if outcomes were mediated through changes in the functional activity of the gut microbiota. This study complements the growing body of clinical data that supports the safety, tolerability, and potential functional benefit bMFGM to infant formula by extending foundational understanding to the earliest period of infant nutrition and microbiota development.

## Data Availability

The datasets presented in this study can be found in online repositories. The names of the repository/repositories and accession number(s) can be found below: https://www.ncbi.nlm.nih.gov/bioproject/1005334, PRJNA1005334.

## Footnotes

1
https://clinicaltrials.gov/ct2/show/

2
https://www.ncbi.nlm.nih.gov/bioproject/1005334
