# Supplementary material for: Microbiota, metabolic profiles and immune biomarkers in infants receiving formula with added bovine milk fat globule membrane: a randomized, controlled trial
Source: Front Nutr. 2024 Oct 4;11:1465174. doi: 10.3389/fnut.2024.1465174 (PMC11497130; doi:10.3389/fnut.2024.1465174)
Supplement: Supplementary file 1 [file Data_Sheet_1.PDF]

List of Supplementary Tables and Figures:

Supplementary Table 1. Consort 2010 Checklist

Supplementary Table 2. Inclusion and exclusion criteria.

Supplementary Table 3. Days of age at baseline across study groups.

Supplementary Table 4. Stool characteristics at Baseline, Day 30, and Day 60.

Supplementary Figure 1: Differences in subject age at baseline.

Supplementary Figure 2: Differences in community composition between study groups and sample sites.

Supplementary Figure 3: Conserved ASVs between stool and oral microbiome.

Supplementary Figure 4: Infant stool microbiota composition at genus level.

Supplementary Figure 5: Infant oral microbiota composition at genus level.

Supplementary Figure 6: Differential heat trees comparing infant oral taxa.

Supplementary Figure 7: Analysis of differentially abundant infant oral ASVs.

Supplementary Table 1: CONSORT 2010 checklist of information to include when reporting a randomised trial*

Section/Topic	Item No	Checklist item	Reported on page No
**Title and abstract**			
	1a	Identification as a randomised trial in the title	1
	1b	Structured summary of trial design, methods, results, and conclusions (for specific guidance see CONSORT for abstracts)	3
**Introduction**			
Background and objectives	2a	Scientific background and explanation of rationale	4-5
	2b	Specific objectives or hypotheses	5
**Methods**			
Trial design	3a	Description of trial design (such as parallel, factorial) including allocation ratio	5-6
	3b	Important changes to methods after trial commencement (such as eligibility criteria), with reasons	NA
Participants	4a	Eligibility criteria for participants	5; Suplementary Table 2
	4b	Settings and locations where the data were collected	5
Interventions	5	The interventions for each group with sufficient details to allow replication, including how and when they were actually administered	5-6
Outcomes	6a	Completely defined pre-specified primary and secondary outcome measures, including how and when they were assessed	6-7
	6b	Any changes to trial outcomes after the trial commenced, with reasons	NA
Sample size	7a	How sample size was determined	9
	7b	When applicable, explanation of any interim analyses and stopping guidelines	NA
Randomisation:			
Sequence generation	8a	Method used to generate the random allocation sequence	6
	8b	Type of randomisation; details of any restriction (such as blocking and block size)	6
Allocation concealment mechanism	9	Mechanism used to implement the random allocation sequence (such as sequentially numbered containers), describing any steps taken to conceal the sequence until interventions were assigned	6

Implementation	10	Who generated the random allocation sequence, who enrolled participants, and who assigned participants to interventions	6
Blinding	11a	If done, who was blinded after assignment to interventions (for example, participants, care providers, those assessing outcomes) and how	6
	11b	If relevant, description of the similarity of interventions	5-6
Statistical methods	12a	Statistical methods used to compare groups for primary and secondary outcomes	9-11
	12b	Methods for additional analyses, such as subgroup analyses and adjusted analyses	9-11
**Results**			
Participant flow (a diagram is strongly recommended)	13a	For each group, the numbers of participants who were randomly assigned, received intended treatment, and were analysed for the primary outcome	9, Figure 1
	13b	For each group, losses and exclusions after randomisation, together with reasons	Figure 1
Recruitment	14a	Dates defining the periods of recruitment and follow-up	5-6
	14b	Why the trial ended or was stopped	9
Baseline data	15	A table showing baseline demographic and clinical characteristics for each group	Table 2
Numbers analysed	16	For each group, number of participants (denominator) included in each analysis and whether the analysis was by original assigned groups	Figure 1; 11-12
Outcomes and estimation	17a	For each primary and secondary outcome, results for each group, and the estimated effect size and its precision (such as 95% confidence interval)	11-16
	17b	For binary outcomes, presentation of both absolute and relative effect sizes is recommended	NA
Ancillary analyses	18	Results of any other analyses performed, including subgroup analyses and adjusted analyses, distinguishing pre-specified from exploratory	11-16
Harms	19	All important harms or unintended effects in each group (for specific guidance see CONSORT for harms)	12
**Discussion**			
Limitations	20	Trial limitations, addressing sources of potential bias, imprecision, and, if relevant, multiplicity of analyses	19-20
Generalisability	21	Generalisability (external validity, applicability) of the trial findings	16-20
Interpretation	22	Interpretation consistent with results, balancing benefits and harms, and considering other relevant evidence	16-20
**Other information**			
Registration	23	Registration number and name of trial registry	3,8
Protocol	24	Where the full trial protocol can be accessed, if available	NA
Funding	25	Sources of funding and other support (such as supply of drugs), role of funders	21

Supplementary Table 2. Inclusion and exclusion criteria.

Inclusion Criteria	Exclusion Criteria
• 7-18 days of age at Visit 1, inclusive (day of birth is considered day 0)<br>• Singleton birth<br>• Gestational age of 37-42 weeks (36 weeks and six days is considered 36 weeks gestational age)<br>• Birth weight of 2500 g (5 lbs 8 oz) or more<br>• Parent or legal guardian agrees not to enroll infant in another interventional clinical study while participating in this study<br>• Signed informed consent obtained from parent or legal guardian for infant's participation in the study<br>• Signed authorization obtained from parent or legal guardian to use and/or disclose protected health information for infant from birth through the length of the study period<br>• Signed authorization obtained from infant's birth mother to use and/or disclose her protected health information from 48 hours prior to infant's birth to randomization/ registration<br>• Receipt of two protocol-compliant Baseline stool samples[†]<br>**Infants receiving formula:**<br>• Exclusively receiving infant formula for at least 24 hours prior to randomization<br>• Parent(s) or legal guardian has full intention to exclusively feed study formula during the study period<br>**Infants receiving human milk:**<br>• Mother has intention to exclusively provide mother's-own breast milk for the duration of the study<br>• Signed authorization obtained from infant's mother to use and/or disclose her protected health information from registration through the length of the study period	• Caesarean delivery<br>• Infant consumption of donor milk prior to randomization/registration<br>• Maternal antibiotic use within 48 hours prior to or at time of delivery (however, topical antibiotic use on any area other than the breast or perineal areas is allowed)<br>• Maternal antibiotic use while providing mother's-own breast milk to infant (however, topical antibiotic use on any area other than the breast area is allowed)<br>• Infant use of systemic antibiotics prior to randomization/registration<br>• Any signs of an acute infection (i.e. fever, diarrhea) at randomization/registration<br>• Infant has been weighed by a health care professional (HCP) and is identified with inadequate weight gain or failure-to-thrive*<br>• Infant use of probiotics (including infant formula with probiotics) prior to enrollment or planned use of probiotics during the study period<br>• Evidence of significant feeding difficulties (from breast or bottle) or history of formula intolerance, such as vomiting or poor intake at time of randomization/registration<br>• Infant was born large for gestational age (LGA) (as confirmed by the hospital birth records) from mother who was diabetic at childbirth<br>• History of underlying metabolic or chronic disease; congenital malformation; or any other condition which, in the opinion of the Investigator, is likely to interfere with: the ability of the infant to ingest food, the normal growth and development of the infant, or the evaluation of the infant<br>• Infant is immunocompromised (according to a doctor's diagnosis of immunodeficiency such as combined immunodeficiencies, DiGeorge syndrome, Wiskott-Aldrich syndrome, severe congenital neutropenia and secondary immunodeficiencies linked to HIV infection, Down syndrome or others)<br>**Infants receiving human milk:**<br>• Consumption of infant formula from 1 day of age to study registration (day of birth is considered day 0)

*amended due to COVID-19 in-person visit restrictions (original criterion: Weight at Visit 1 is <95% of birth weight [(weight at Visit 1÷ birth weight) x 100 <95%])

[†] reduced from three total samples

Supplementary Table 3. Days of age at baseline across study groups.

	Median	25th percentile	75th percentile	Range	p
INV-MFGM	14.50	9.75	14.25	4-15	0.3232
Control	13.00	7.00	14.00	5-16	
HM	13.00	9.75	16.00	9-16	

Supplementary Table 4. Stool consistency at Baseline, Day 30, and Day 60.

		Stool consistency, n (%)					
Day	Group	hard	formed	mushy	unformed or seedy	watery	p
Baseline	Control	0 (0)	1 (6)	7 (39)	10 (56)	0 (0)	0.269
	INV-MFGM	0 (0)	0 (0)	11 (58)	8 (42)	0 (0)	
	HM	0 (0)	0 (0)	5 (29)	12 (71)	0 (0)	
30	Control	0 (0)	0 (0)	10 (83)	2 (17)	0 (0)	0.047[a]
	INV-MFGM	0 (0)	0 (0)	11 (73)	4 (27)	0 (0)	
	HM	0 (0)	0 (0)	5 (38)	8 (62)	0 (0)	
60	Control	0 (0)	0 (0)	10 (83)	2 (17)	0 (0)	0.004[a,b]
	INV-MFGM	0 (0)	0 (0)	11 (79)	3 (21)	0 (0)	
	HM	0 (0)	0 (0)	2 (25)	4 (50)	2 (25)	

[a]Control vs. HM significantly different ($p<0.05$).
[b]INV-MFGM vs. HM significantly different (<0.05).

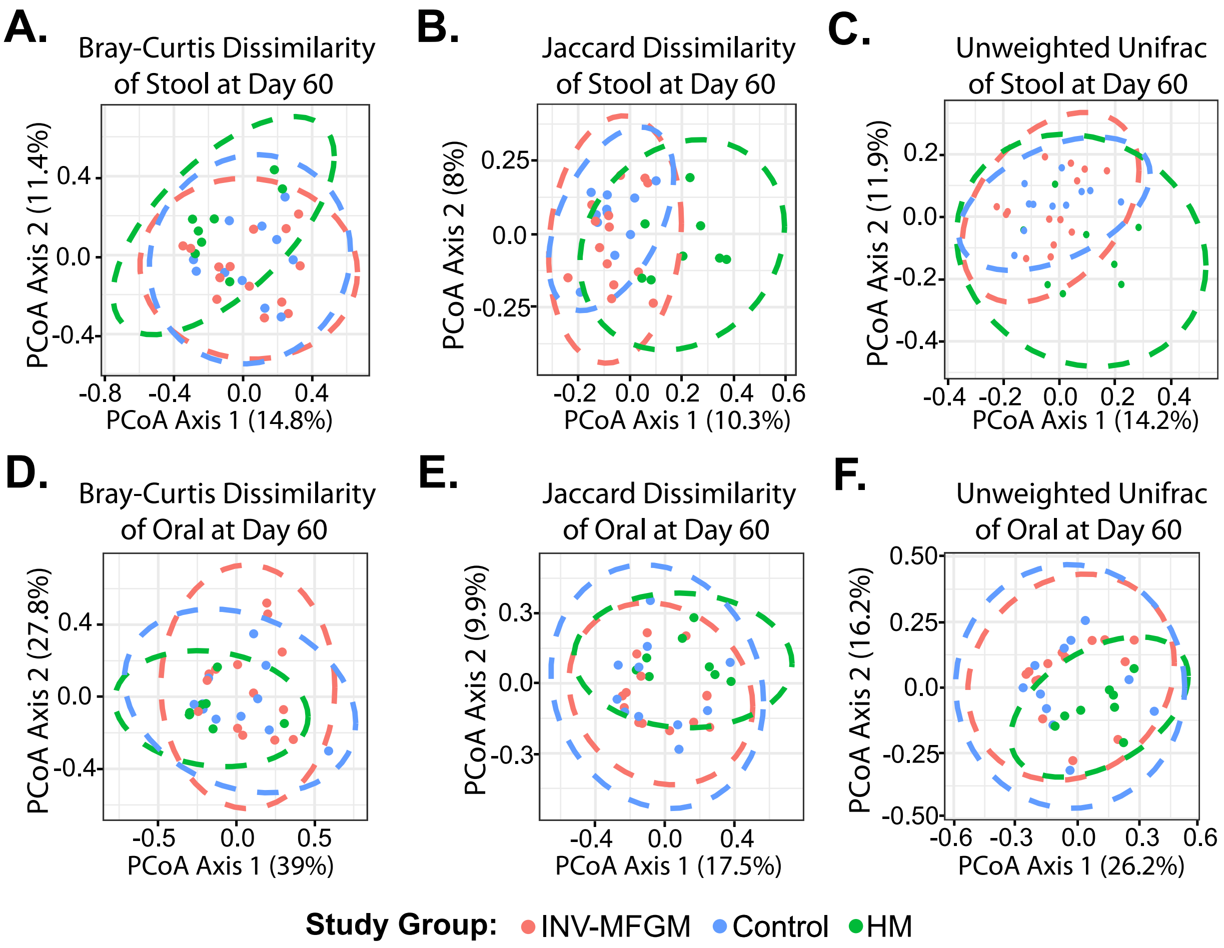

Supplementary Figure 1. Differences in community composition between study groups at Day 60. Bray-Curtis (A,D), Jaccard (B,E), and Unweighted Unifrac (C,F) dissimilarity values were plotted and significance of differences were tested by PERMANOVA for stool samples (A-C) and oral samples (D-F). (A) Statistically significant comparison included INV-MFGM vs HM: R^2=0.0828, p=0.030 (B) Statistically significant comparisons included INV-MFGM vs HM: R^2=0.0886, p=0.003 and Control vs HM: R^2=0.120, p=0.003. (C) No statistically significant differences. (D) Statistically significant comparisons included INV-MFGM vs HM: R^2=0.1334, p=0.039. (E)-(F). No statistically significant differences.

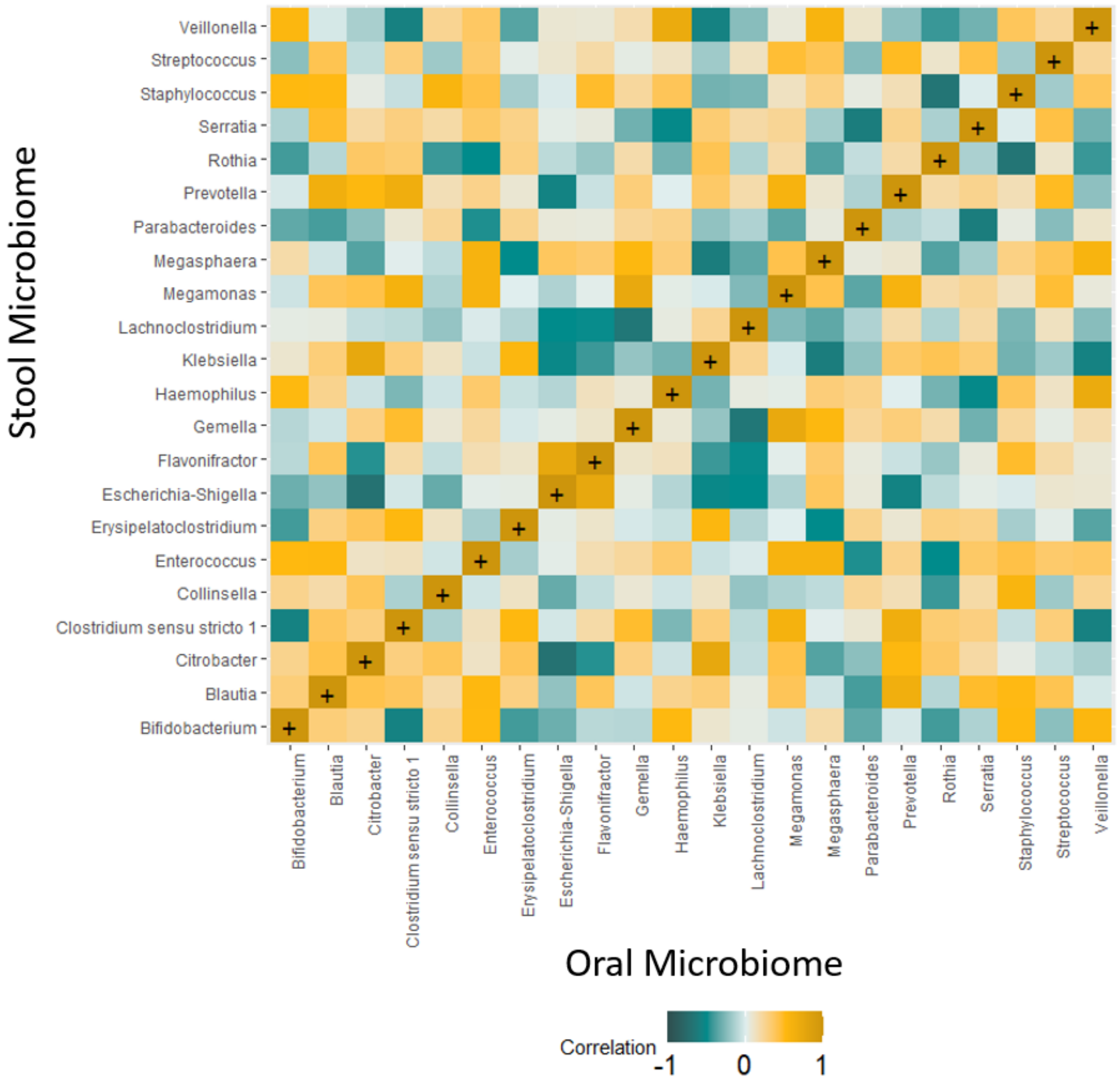

Supplementary Figure 2: Conserved ASVs between stool and oral microbiome. Spearman correlations of stool bacterial taxa with oral bacterial ASVs. To remove any possible noise, an abundance threshold of 0.25% was utilized. + indicates significant correlation and conserved taxa presence above the abundance threshold (FDR < 0.05). The genus-level classification for each ASV is provided.

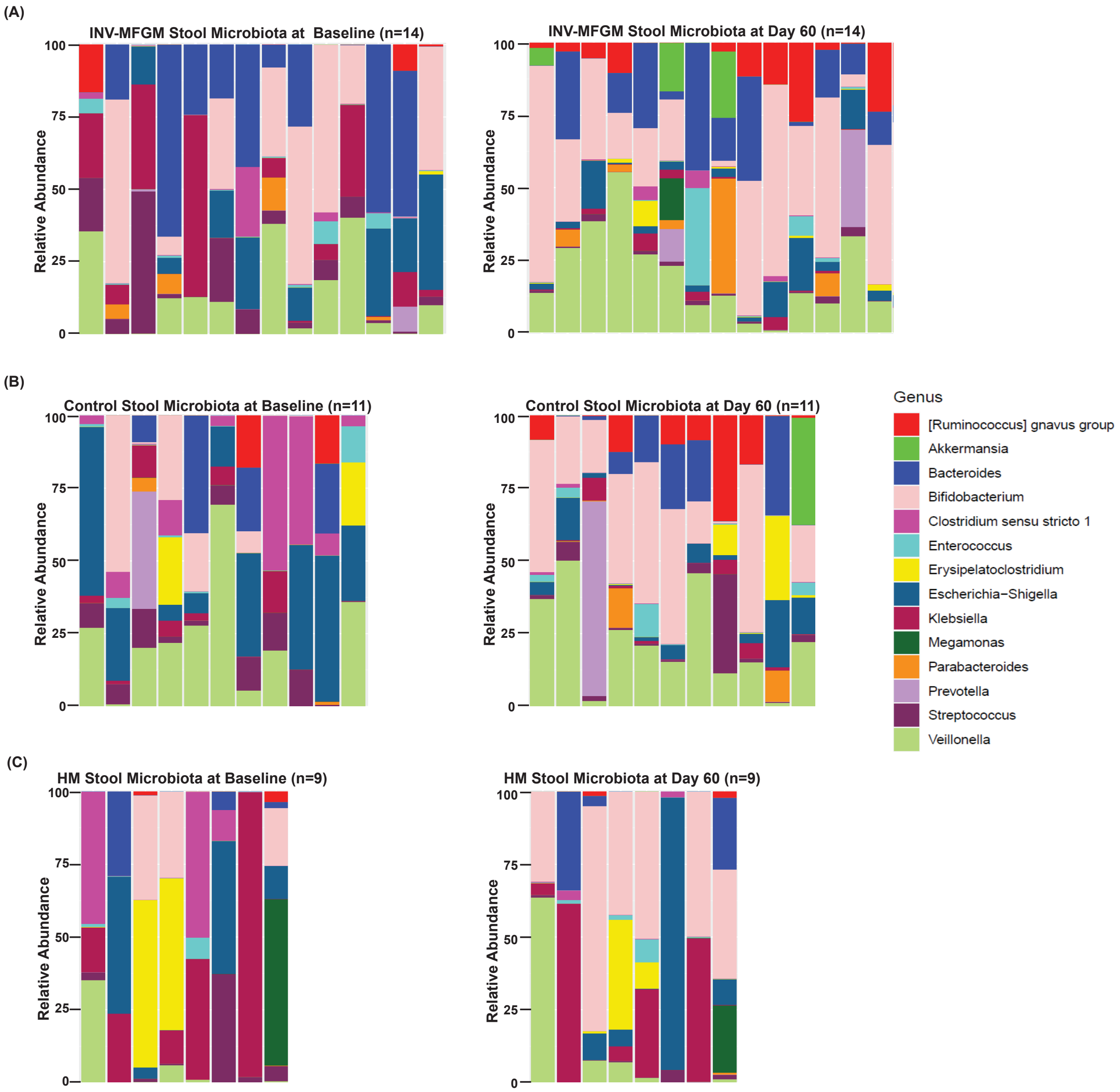

Supplementary Figure 3: Infant stool microbiota composition at genus level. Genus-level taxonomic composition of stool microbiota for infants in (**A**) INV-MFGM, (**B**) Control, or (**C**) HM study groups at Baseline and Day 60. Each column represents an individual subject within that study group, with subject order maintained across Baseline and Day 60 graphs. The relative abundance of all genera that met a threshold of >0.1 was shown.

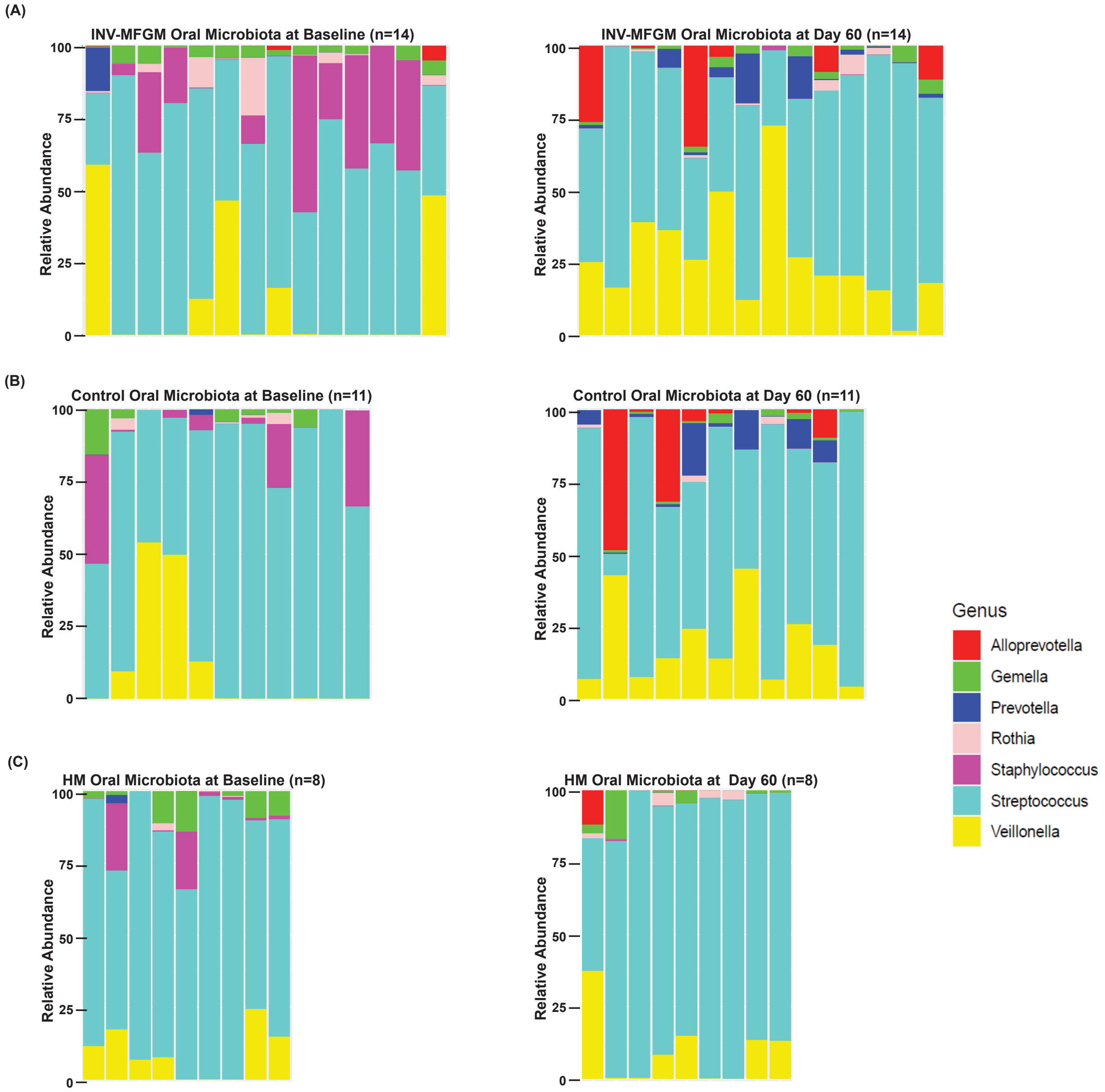

Supplementary Figure 4: Infant oral microbiota composition at genus level. Genus-level taxonomic composition of stool microbiota for infants in (**A**) INV-MFGM, (**B**) Control, or (**C**) HM study groups at Baseline and Day 60. Each column represents an individual subject within that study group, with subject order maintained across Baseline and Day 60 graphs. The relative abundance of all genera that met a threshold of >0.1 was shown.

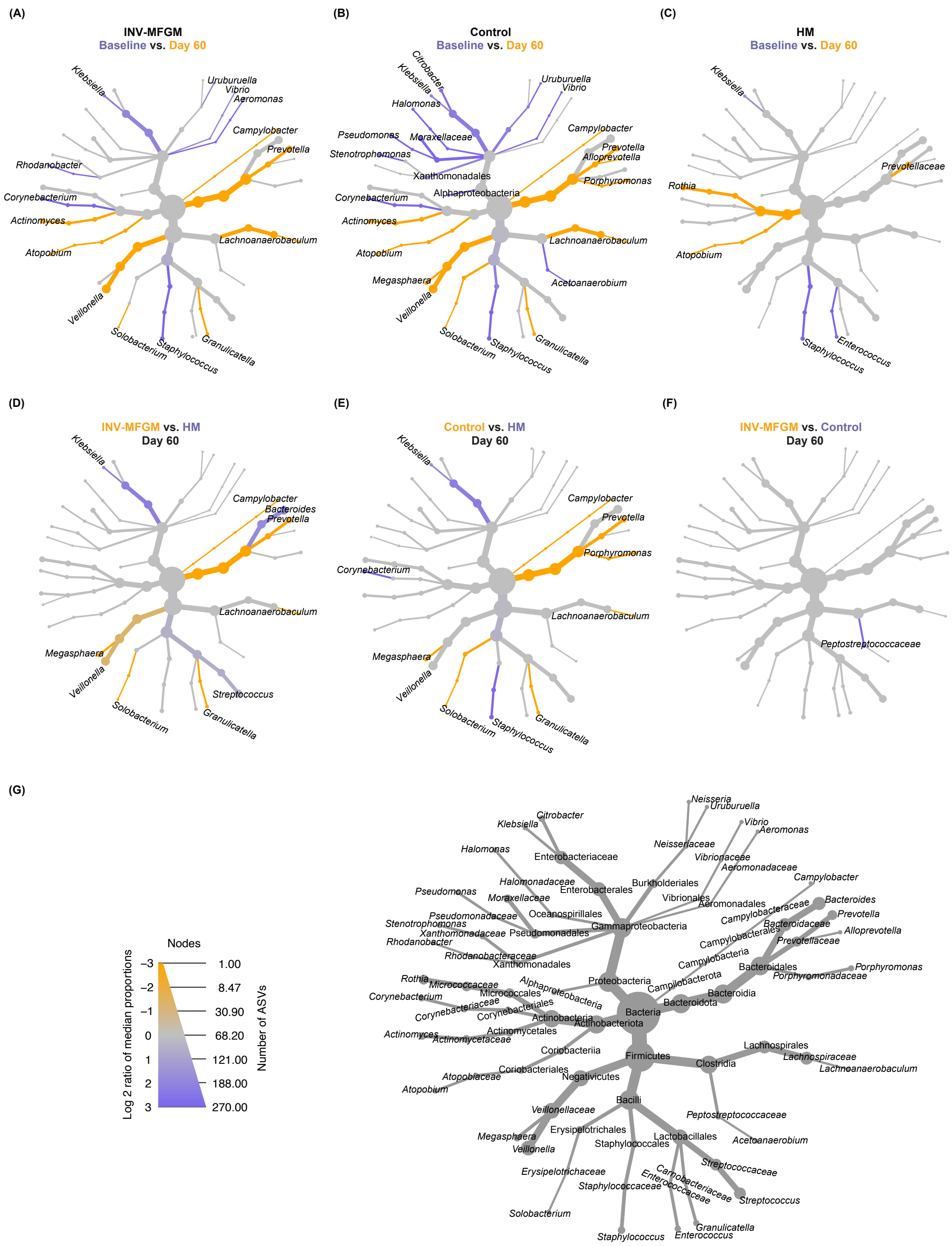

Supplementary Figure 5: Differential heat trees comparing infant oral taxa. Difference in relative abundance by study time point (Baseline vs. Day 60) were shown by feeding group: (**A**) INV-MFGM, (**B**) Control, and (**C**) HM. Differences in relative abundance between study groups at Day 60 were shown by comparisons of: (**D**)INV-MFGM vs HM, (**E**)Control vs HM, and (**F**)INV-MFGM vs Control. (**G**) A reference taxonomic tree representing all taxa with differential abundance and key indicating the correlations between node size and total ASVs and color intensity with relative abundance.

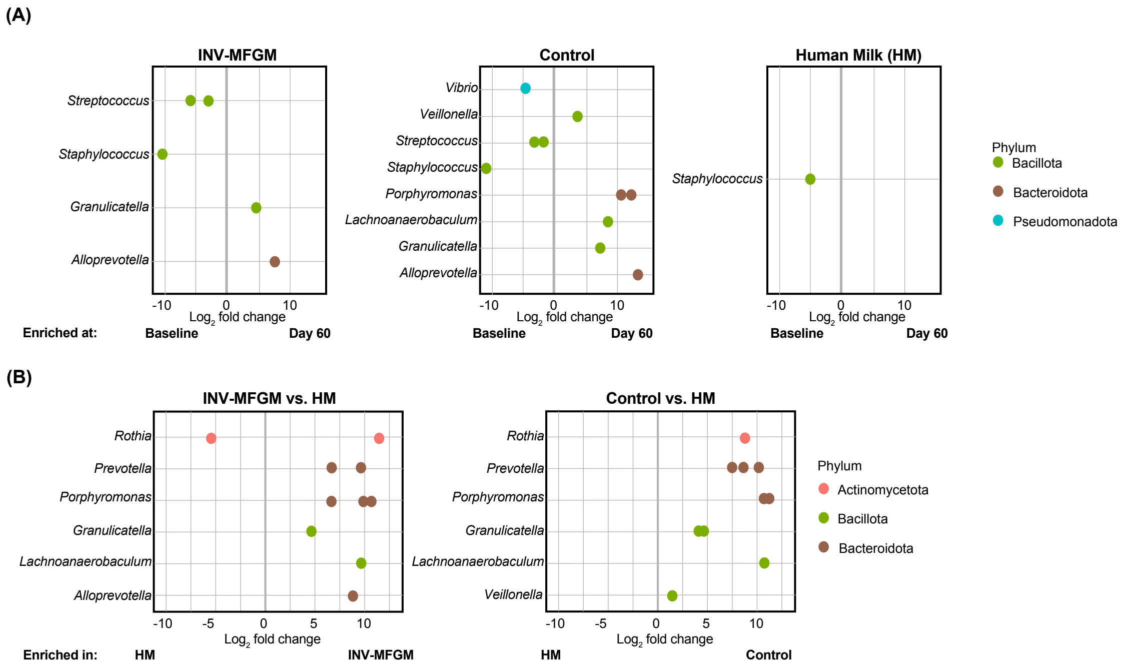

Supplementary Fig. 6: Analysis of differentially abundant infant oral ASVs. DESeq2 was used to identify ASVs with significantly different abundance (FDR <0.05) between (**A**) Baseline and Day 60 time points and (**B**) study groups at Day 60. Study groups are indicated above the plots. Only ASVs with mean reads > 100 are shown.
